# Effect of Collagen Cross-Linking on Alkali Burn-Induced Corneal Neovascularization in Rabbits

**DOI:** 10.1155/2018/7325483

**Published:** 2018-10-09

**Authors:** Xiaoying Xu, Taixiang Liu, Haixiang Li

**Affiliations:** ^1^Zunyi Medical College, Zunyi 563003, China; ^2^Guizhou Ophthalmic Hospital, The Affiliated Hospital of Zunyi Medical College, Zunyi 563003, China

## Abstract

**Objective:**

This study aims at investigating the effects and molecular mechanism of riboflavin-ultraviolet-A-induced cross-linking (corneal collagen cross-linking, CXL) on corneal neovascularization (CNV) in a rabbit alkali burn model.

**Methods:**

A total of 60 rabbits were injured with alkali burns to induce CNV in the right eye and were randomly divided into six groups: Group A—injury and no treatment; Groups B, C, and D—CXL treatment for 30 min, 15 min, and 45 min administered immediately after injury, respectively; and Groups E and F—CXL treatment for 30 min administered 1 day and 3 days after injury, respectively. CNV area, corneal edema, and corneal epithelial defects were observed on days 4, 7, 10, and 14 after injury. Western blot was used to detect expression of the vascular endothelial growth factor (VEGF), matrix metalloproteinase-2 (MMP-2), matrix metalloproteinase-2 (MMP-9), and tissue inhibitor of metalloproteinases 1 (TIMP-1) at 7 and 14 days after injury.

**Results:**

CXL treatment decreased CNV and corneal edema in all groups compared to Group A. On day 7, MMP-9 expression was significantly increased in all CXL treatment groups, and TIMP-1 was upregulated in Groups D and F compared to Group A. In addition, VEGF, MMP-2, MMP-9, and TIMP-1 expression were increased in Group A on day 14 after injury.

**Conclusions:**

Our results indicate that riboflavin-ultraviolet-A-induced cross-linking (corneal collagen cross-linking, CXL) significantly inhibits alkali burn-induced CNV in rabbits, possibly through downregulating VEGF, MMP-2, MMP-9, and TIMP-1 expression.

## 1. Introduction

Alkali burns are considered an ophthalmologic emergency that can induce corneal neovascularization (CNV) associated with corneal edema, increased secretion of angiogenic factors, inflammatory response, and hypoxia [1]. Previous studies indicated that the vascular endothelial growth factor (VEGF) and matrix metalloproteinases (MMPs) stimulate CNV after chemical burns by degrading the vascular basement membrane. Several other molecules also play an antiangiogenic role, including endostatin, angiostatin, and tissue inhibitors of metalloproteinases (TIMPs).

As previously demonstrated, cross-linked collagen type I and type IV can resist cleavage by MMP-1, MMP-2, MMP-9, MMP-13, and noncross-linked collagen type I and type IV. However, glycosylated small leucine-rich proteoglycans (SLRPs) are susceptible to degradation by MMPs [2]. Riboflavin-ultraviolet-A corneal cross-linking (CXL) is an established treatment to enhance biomechanical stability, increase corneal resistance to enzymatic digestion, and inhibit corneal edema [3–5]. Moreover, in a corneal alkali burn model, epithelial defects are attenuated by CXL treatment, which suggests that CXL ameliorates the effects of acute corneal alkali burns [6]. In the present study, we investigated the pathogenic process of alkali burn-induced CNV with or without riboflavin-ultraviolet-A-induced cross-linking and measured the expression of VEGF, MMP-2, and MMP-9 to determine the molecular mechanism of CXL for promoting angiogenesis.

## 2. Materials and Methods

### 2.1. Animals and Grouping

A total of 60 New Zealand rabbits (2.0–2.5 kg) were purchased from Zunyi Laboratorial Animal Center, Chinese Academy of Sciences and randomly separated into six groups: Group A was injured with alkali burns to induce CNV and given no treatment; Groups B, C, and D were administered CXL for 30 min, 15 min, and 45 min immediately after injury, respectively; and Groups E and F were administered CXL for 30 min after 1 day and 3 days injury, respectively. The right eye was chosen to induce CNV. All animal experiments were conducted according to the Association for Research in Vision and Ophthalmology (ARVO) Statement for the Use of Animals in Ophthalmic and Vision Research.

### 2.2. Establishment of Alkali Burn-Induced Corneal Injury Model and Induction of CNV

The animal model of CNV was established as described previously [7]. In brief, rabbits were anesthetized by an intravenous injection of 3% sodium pentobarbital (1 ml/kg), and topical ocular anesthesia was induced by 0.5% proparacaine hydrochloride ophthalmic drops in the animals' right eyes. Under a surgical microscope, an 8 mm diameter filter paper soaked in alkali (1 mol/L) for 10–20 s was applied to the cornea of the right eye. A drop of 25 *μ*L alkali was applied to the filter paper after 1 min to maintain contact with the cornea. Two minutes after the injury procedure, eyes were rinsed with 15 ml of 0.9% NaCl. Then, 0.1% riboflavin drops (dissolved in 20% dextran T500) were applied every 2–5 min for 30 min in the experimental groups. Irradiation with ultraviolet light (UVA 370 ± 5 nm, with an irradiance of 3 mW/cm^2^ and a surface dose of 5.4 J/cm^2^) followed for 30 min, 15 min, and 45 min in Groups A, B, and C, respectively. During UVA irradiation, we continued to apply 0.1% riboflavin drops to the total cornea and limbus area concomitantly every 3 min.

### 2.3. CNV Assessment

The corneal surface was photographed on days 4, 7, 10, and 14 after alkali burn by microscopy using a hand-held slit lamp. The following formula was used to calculate the CNV area: area (mm^2^) = *C*/12 × 3.1416 × [R^2^-(R-L)^2^] [8], where *C* is the clock hour of neovascularization (1 clock hour equals 30 degrees of arc), *R* is the radius of the cornea, (approximately 7 mm in rabbits), and *L* is the maximal vessel length, extending from the limbal vasculature.

### 2.4. Western Blot Analysis

Five rabbits were sacrificed on days 7 and 14, respectively, and the corneal tissues were excised and homogenized using immunoprecipitation assay (RIPA) lysis buffer. VEGF, MMP-2, MMP-9, and TIMP-1 protein levels were measured by western blot analysis. Proteins from whole lysates (10 *μ*g protein per sample) were electrophoresed by 7.5% SDS-PAGE and then electroblotted onto nitrocellulose membranes that were blocked in 5% skim milk in Tris-buffered saline containing 0.05% Tween (TBST) buffer for 1 h at room temperature. After rinsing with TBST, the membranes were incubated with antibodies against rat VEGF, MMP-2, MMP-9, and TIMP-1 (Boaoseng, Beijing, China, all at 1 : 500 in TBST). Following three washes with TBST, the blots were further incubated with a horseradish peroxidase-conjugated secondary antibody for 1.5 h at room temperature. Chemiluminescence assays were processed using a peroxidase substrate. The immunoblot signal was detected and analyzed using Image J software.

### 2.5. Statistical Analysis

CNV area was analyzed using a two-way repeated measures ANOVA using SPSS Statistics 17.0. Measurements of VEGF, MMP-2, MMP-9, and TIMP-1 were statistically analyzed using a one-way analysis of variance (ANOVA) test. LSD-t comparisons were used to isolate significant interactions. A value of *P* < 0.05 was considered statistically significant.

## 3. Results

### 3.1. Evaluation of the Corneal Surface

**Figure 1:**
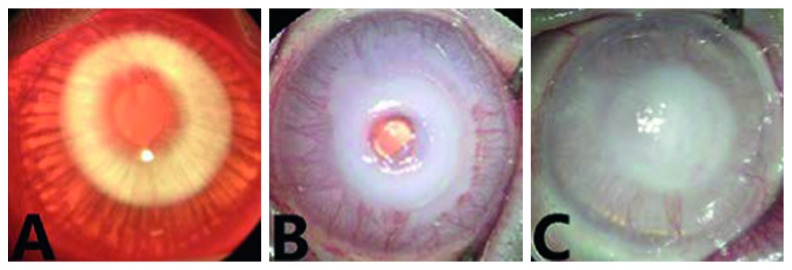
Corneal morphology in rabbits: (a) normal ocular surface; (b) ocular surface with alkali burn; (c) ocular surface with alkali burn and CXL treatment.

Examinations were performed 3 days after alkali burn injury. The ocular surface was transparent and smooth with no blood vessels, and there was a clear iris texture and pupil (Figure 1). There was a white round turbid edema area about 8 mm in the central cornea, and conjunctivitis was observed in the alkali burn group (Figure 1). Moreover, the central matrix was dissolved in the cornea of 5 eyes (50%), and the central cornea was thinned with no corneal perforation in a total of 7 eyes (14%) in the alkali burn group.

### 3.2. CNV Assessment

Blood vessel buds started growing 3 days after alkali burn injury, spreading was fastest from the limbus into nearly the whole corneal stroma (during 7–14 days after injury) with more than five branches, and secretion was visible in the conjunctiva and on the eyelid margins. The CNV area displayed less neovascularization compared to the normal group (Figure 1 and Table 1).

**Figure 2:**
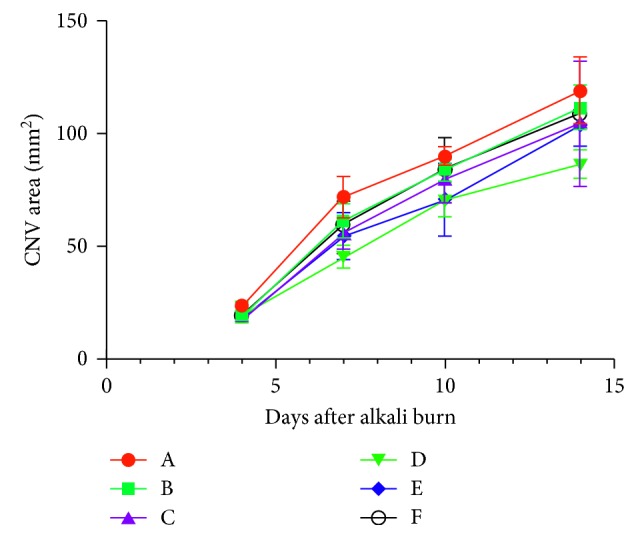
CNV area at different times.

A two-way repeated measures ANOVA was conducted since there were 6 groups with a dependent variable of four time points. The results indicate that CXL treatment significantly attenuated alkali burn-induced CNV on days 4, 7, 10, and 14 in Groups B, C, D, E, and F compared to Group A. However, CNV increased in a time-dependent manner in all groups (Figure 2). There were no obvious changes in the CNV area in any of the Groups on day 4. However, on day 7, after alkali injury, the CNV area was significantly reduced in Groups B, C, D, E, and F, especially in Group D, which displayed the smallest CNV area (*P* < 0.05). Groups D and E displayed significantly less neovascularization compared to Group A on day 10 after injury. Interestingly, the CNV area in Group D was smaller compared to Groups A, B, and F, which suggest that 45 min CXL effectively and stably inhibited CNV induced by alkali burn in a time-dependent manner.

### 3.3. VEGF, MMP-2, MMP-9, and TIMP-1 Expression after CXL Treatment

We investigated the mechanism by which CXL decreases corneal CNV after alkali burns by performing western blot analysis of corneal VEGF, MMP-2, MMP-9, and TIMP-1 expression. MMP-9 expression was increased on day 7 after alkali injury in Groups B, C, D, E, and F in response to CXL treatment (*P*=0.000, 0.000, 0.023, 0.015, and  0.001, resp.) compared to Group A. TIMP-1 expression was significantly higher in Groups D and F compared to Group A (*P*=0.018  and  0.004).

**Figure 3:**
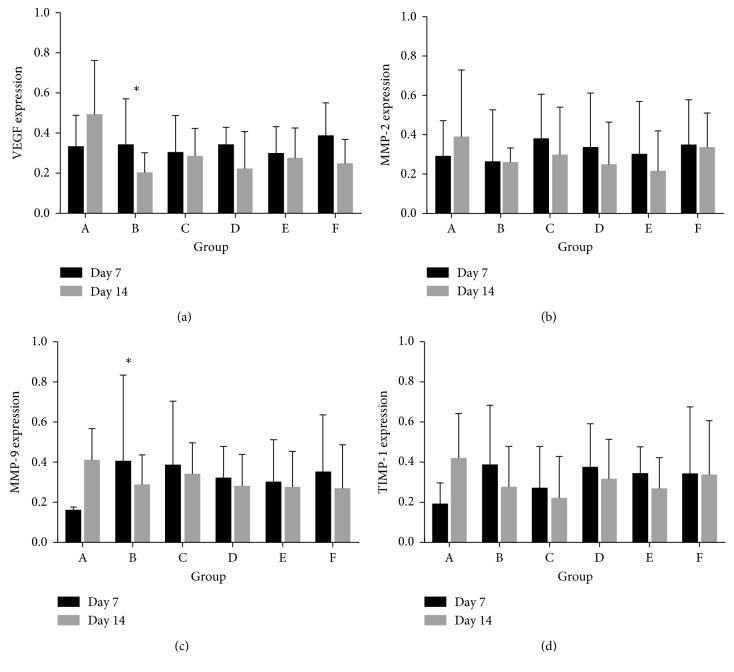
Effect of CXL on VEGF, MMP-2, MMP-9, and TIMP-1 expression on days 7 and 14 after corneal alkali burns.

As shown in Table 2, VEGF expression was significantly lower in all treatment groups compared to Group A (Group B: *P*=0.003; Group C: *P*=0.026; Group D: *P*=0.014; Group E: *P*=0.025; and Group F: *P*=0.009). MMP-2 was significantly decreased in treatment Groups B, D, and E compared to Group A (Group B: *P*=0.001; Group D: *P*=0.018; and Group E: *P*=0.006). There was no significant difference in the expression of MMP-9 and TIMP-1 in all treatment groups compared to Group A (Figure 3).

## 4. Discussion

Corneas remain transparent due to various structural and metabolic adaptations. However, CNV induced by chemical exposure has been identified as a pathological inducer of blood vessel growth in the cornea, which leads to loss of visual function. Further, CNV is a high-risk factor for corneal allograft transplantation due to damage to the entire anterior segment of the eye. Currently, 20% of corneal diseases result from CNV induced by ocular trauma.

VEGF is considered a primary mediator of CNV, which results from a disruption in the balance of angiogenic and antiangiogenic factors. VEGF stimulates vascular permeability and acts as a mitogen and angiogenic factor in endothelial cells. VEGF-A is one of the main regulators of angiogenesis, which is upregulated during neovascularization. Upregulation of VEGF can be induced through corneal injury, and neovascularization can be subsequently blocked by anti-VEGF antibodies. As previously demonstrated, the anti-VEGF agent, bevacizumab, inhibits interactions between VEGF and its receptors and has been recently applied to treat various neovascular ocular diseases [9]. Indeed, bevacizumab has been shown to effectively reduce CNV in both animal models and clinical trials [10].

MMPs are a group of enzymes that degrade extracellular matrix proteins (ECM). Numerous studies support a causal role for MMPs in CNV. MMP-2 and MMP-9 have been implicated in the development of neovascularization. Expression of both MMPs increases during CNV. Previous studies indicated that an increase in membrane type-1 matrix metalloproteinase- (MT1-) MMP increases VEGF production, suggesting that MMPs facilitate CNV. MT1-MMP has been shown to enhance the basic fibroblast growth factor- (bFGF-) induced CNV *in vivo* [11]. Ethanol extract of *Diospyros kaki* (EEDK) decreased the CNV area compared to a control group, which was partially attributed to the downregulation of VEGF, FGF, interleukin-6 (IL-6), and MMP-2 protein levels [12].

The photosensitizer riboflavin can penetrate the corneal stroma and be excited by UVA to create free radicals, which results in collagen cross-linking [13]. Corneal collagen cross-linking (CXL) therapy, administered as a combination of riboflavin (vitamin B2) and UVA light, can enhance corneal biomechanical stability and promote resistance to enzymatic digestion. CXL can also significantly increase corneal stiffness and decrease damping capability and deformability [14–16].

The ultraviolet-visible light transmission spectrum was 10–20% lower in cross-linked corneas compared to control corneas, which conferred CXL's protection against ultraviolet penetration [17].

CXL has been used to treat different kinds of corneal disorders, such as neovascular glaucoma, keratitis, and bullous keratopathy. Seven eyes from 6 patients with severe infectious keratitis improved after CXL, which indicated that CXL is an effective tool for treating infectious keratitis [18]. Massive corneal edema can cause opacities, usually due to the disruption of the corneal stroma architecture, and CXL can resolve the edematous condition, improving both corneal transparency and visual acuity. Thus, CXL is a useful method to decrease edematous conditions and improve corneal transparency and visual acuity [19].

In the present study, we found that the central corneal matrix was dissolved in the corneas of 5 eyes (50%), while only 7 eyes (14%) displayed thinning in the central corneal with no corneal perforation following alkali burn injury. These results indicate that CXL induced resistance to enzymatic digestion, as reported in previous studies. CXL treatment also changed the morphology of stromal collagen, as evidenced by second harmonic generation imaging. These data showed wavier morphology in the linear collagen fibers. Our biochemical analyses also indicate that CXL treatment causes collagen cross-linking not only in the anterior region of the cornea but also more deeply (>200 *µ*m from the anterior region) [20].

Our results showed that the area of alkali burn-induced CNV was significantly decreased on days 4, 7, 10, and 14 following CXL treatment, especially in Group D which had the smallest CNV area. CXL can increase corneal stiffness by inducing cross-links within the ECM. Previous studies reported that different exposure times could have different biomechanical effects of riboflavin-UVA irradiation-induced CXL [21]. A total of 30 min CXL treatment with riboflavin increased corneal tissue stiffness. The current study shows that 45 min riboflavin-UVA CXL treatment most effectively reduced CNV, which supports previous reports.

Ultraviolet-activated riboflavin treatment can inhibit the MMP-9 activity to increase immediate bond strength and stabilize the adhesive interface [22]. CXL treatment significantly elevated MMP-13 in the tear fluid of keratoconus patients, which was partially attributed to the active form of the tissue plasminogen activator that activates the MMP pathway [23]. Further studies are needed to investigate the role of CXL on MMPs. Our results show that CXL decreased VEGF, MMP-2, MMP-9, and TIMP-1 expression after alkali injury over time. MMP-9 expression was increased on day 7 in Groups B, C, D, E, and F in response to CXL treatment, and TIMP-1 expression was significantly higher in Groups D and F compared to Group A. On day 14 after alkali injury, MMP-2 and VEGF expression were significantly lower in the CXL treatment groups compared to the control group. However, there was no significant difference in the expression of MMP-9 and TIMP-1 in any of the treatment groups. Together, our results suggest that MMP-9 and TIMP-1, and VEGF and MMP-2, have different functions in CNV following CXL treatment. TIMPs have been characterized as endogenous inhibitors of MMPs. Disruption of the MMP/TIMP balance may be associated with corneal inflammation after alkali injury. MMP-9 expression inversely correlated with TIMP-1 28 days after alkali burns in rabbits [24]. Our results show that TIMP-1 expression increased on day 7 compared to the control group and decreased by day 14 after injury. More studies are needed to investigate the signaling pathway mediated by CXL.

Collectively, our study further verifies that CXL effectively reduces the CNV area, possibly via downregulating VEGF, MMP-2, and MMP-9 expression. Further studies will be conducted to investigate the mechanism by which CXL treatment of alkali injury induces CNV.

## Figures and Tables

**Table 1:** CNV area at different time points.

Group	The CNV area（$\bar{x}$ ± *s*, mm^2^）				
	*n*	4 days	7 days	10 days	14 days
Group A (alkali burns with treatment)	5	23.24 ± 2.13	69.38 ± 10.01^*∗*^	90.19 ± 4.21	119.22 ± 14.58
Group B (alkali burns with 30 min CXL)	5	20.89 ± 4.99	57.07 ± 9.30^*∗*^	84.17 ± 4.77	111.47 ± 9.84^#^
Group C (alkali burns with 15 min CXL)	5	20.19 ± 4.84	55.30 ± 9.44^*∗*^	79.67 ± 10.55	104.56 ± 27.77
Group D (alkali burns with 45 min CXL)	5	19.77 ± 3.78	53.68 ± 11.98^*∗*^	70.70 ± 7.75^*∗*^	86.33 ± 6.22^*∗*^
Group E (alkali burns 1 day later with 30 min CXL)	5	21.94 ± 5.80	57.19 ± 9.69^*∗*^	70.47 ± 16.06^*∗*^	103.71 ± 9.95
Group F (alkali burns 3 day later with 30 min CXL)	5	19.43 ± 2.74	58.94 ± 8.10^*∗*^	84.11 ± 14.25	109.94 ± 4.01^#^

Two-way repeated measures ANOVA (*F*, *P*)					
Groups	5.515, <0.002				
Time points	545.48, <0.000				
Groups ∗ time points	1.485, 0.134				

LSD-t test was used for pairwise comparison, with significant mark *P* < 0.05: ^*∗*^Comparison with group A. ^*#*^Comparison with group D.

**Table 2:** Effect of CXL on VEGF, MMP-2, MMP-9, and TIMP-1 expression after corneal alkali burns.

Group	*n*	Day 7 after alkali burn				Day 14 after alkali burn			
		VEGF	MMP-2	MMP-9	TIMP-1	VEGF	MMP-2	MMP-9	TIMP-1
A	5	0.44 ± 0.21	0.42 ± 0.16	0.17 ± 0.14	0.27 ± 0.11	0.68 ± 0.29	0.63 ± 0.14	0.52 ± 0.29	0.58 ± 0.26
B	5	0.50 ± 0.17	0.45 ± 0.07	0.71 ± 0.09^*∗*^	0.60 ± 0.17	0.27 ± 0.12^*∗*^	0.31 ± 0.20^*∗*^	0.39 ± 0.17	0.42 ± 0.13
C	5	0.43 ± 0.16	0.54 ± 0.21	0.61 ± 0.15^*∗*^	0.42 ± 0.12	0.38 ± 0.17^*∗*^	0.47 ± 0.12	0.45 ± 0.22	0.37 ± 0.07
D	5	0.40 ± 0.27	0.53 ± 0.13	0.43 ± 0.20^*∗*^	0.53 ± 0.22^*∗*^	0.35 ± 0.08^*∗*^	0.40 ± 0.09^*∗*^	0.39 ± 0.16	0.46 ± 0.17
E	5	0.39 ± 0.19	0.49 ± 0.10	0.45 ± 0.14^*∗*^	0.44 ± 0.24	0.38 ± 0.16^*∗*^	0.36 ± 0.06^*∗*^	0.40 ± 0.14	0.38 ± 0.15
F	5	0.50 ± 0.26	0.51 ± 0.18	0.55 ± 0.14^*∗*^	0.58 ± 0.10^*∗*^	0.33 ± 0.15^*∗*^	0.46 ± 0.20	0.42 ± 0.11	0.53 ± 0.14
F		0.239	0.481	7.751	2.511	2.742	3.004	0.376	1.373
*P*		0.941	0.787	0.00	0.058	0.043	0.03	0.86	0.27

LSD*-t* comparison was conducted. ^*∗*^means *P* < 0.05 compared to Group A.

## Data Availability

The data used to support the findings of this study are included within the article.
